# Supplementary material for: Identification of immune-related biomarkers associated with allergic rhinitis and development of a sample diagnostic model
Source: PLoS One. 2025 Sep 3;20(9):e0329549. doi: 10.1371/journal.pone.0329549 (PMC12407488; doi:10.1371/journal.pone.0329549)
Supplement: Attachment — Submitted filename: Responses to Reviewers.docx [file pone.0329549.s016.docx]

Response to reviewers

Dear Editor and Reviewers,

We sincerely appreciate the time and effort you have dedicated to reviewing our manuscript entitled “***Identification of immune-related biomarkers associated with allergic rhinitis and development of a sample diagnostic model***” (ID: PONE-D-25-10015). Your insightful comments and constructive suggestions have been invaluable in improving the quality and clarity of our work. We have carefully addressed the points raised, and the revisions are highlighted in yellow in the revised manuscript. Below, we provide a detailed response to each reviewer's comments.

**Responses to reviews (original comments by reviews are in blue color)**

**Reviewer # 1:**

**Comment:** This manuscript presents a comprehensive bioinformatics and experimental approach to identify immune-related biomarkers for allergic rhinitis (AR) and to develop a gene-based diagnostic model. The authors successfully integrate transcriptomic data, network analysis, machine learning, and animal model validation. The study is well-structured, methodologically sound, and contributes valuable insights into AR diagnostics and potential therapeutic targets. In my opinion, the manuscript present a well-executed study offering a novel and clinically relevant diagnostic model for AR based on immune-related biomarkers. This manuscript is suitable for publication in is current form.

**Reply:** We sincerely express our gratitude for your acknowledgment and endorsement of our work.

**Reviewer # 2:**

**1. Comment:** Line 33 and 36. sva and limma need full names or nomenclature. What software have been used for the package? Please be specific in the abstract.

**Reply:** We sincerely appreciate your thoughtful review and valuable feedback on our manuscript. In response we have now consistently supplemented the full nomenclature of the bioinformatics tools “sva” (Surrogate Variable Analysis) and “limma” (Linear Models for Microarray Data) in the abstract. Regarding software implementation, we have explicitly specified the utilization of R packages along with their corresponding versions to ensure complete consistency between the Abstract and Methods sections. (The requested revisions have been implemented in **Lines 40-46 of the** **file labeled 'Revised Manuscript with Track Changes',** with all modifications **highlighted in yellow for clarity**).

**2. Comment:** Line 39. SVM and ROC are famous but still need full name at the first time showing up.

**Reply:** Thank you very much for your constructive suggestions. In response, we have thoroughly addressed the requested revisions as follows: In **Lines 49-51 of the** **file labeled 'Revised Manuscript with Track Changes'** (**highlighted in yellow**), we introduced the full terms **"Support Vector Machine (SVM)"** and **"Receiver Operating Characteristic (ROC)"** upon their first appearance. To ensure terminological consistency, we have systematically standardized all technical acronyms throughout the manuscript following the **"Full Name (Acronym)"** format upon initial use, thereby maintaining uniform compliance across all sections.

**3. Comment:** Line 23. Although it is the trend fashion using 'We' and it seems for emphasizing the major 'achievement' of a study in generally abstract and conclusion sections, please apply passive transformation for most part of scientific manuscript.

**Reply:** We sincerely thank the reviewer for their valuable guidance on scientific writing conventions. In accordance with this recommendation, we have systematically transformed active constructions into passive voice throughout the manuscript, paying particular attention to the Abstract and Conclusion sections. These revisions ensure complete adherence to the journal's prescribed style for objective academic discourse while maintaining methodological precision.

**4. Comment:** Line 169. Any ethical review protocol has been applied for the mice experiment?

**Repl**y: Thanks very much for your valuable questions. We confirm full adherence to international standards for the animal research ethics. The study was conducted under ethical approval No, 2025-083 granted by the Animal Experiment Ethics Committee of Yuhuangding Hospital, with all procedures strictly performed in accordance with the approved guidelines. We have provided additional details regarding the animal experiments, including methods of euthanasia and anesthesia, as well as measures implemented to minimize suffering. (The requested revisions have been implemented in **Lines 171-173 and Lines 185-190 of the** **file labeled 'Revised Manuscript with Track Changes',** with all modifications **highlighted in yellow for clarity**).

**5. Comment:** Line 229. 'Error! Reference source not found.' seems something wrong.

**Reply:** We sincerely appreciate your suggestion and apologize for the oversight. We confirm that this issue stemmed from a technical artifact caused by figure-format incompatibility during the initial submission process. The revised manuscript now accurately displays all references, as evidenced at **Line 236 of the** **file labeled 'Revised Manuscript with Track Changes'** (**highlighted in yellow**) and consistently throughout the document, with all formatting errors fully resolved.

**6. Comment:** Line 254. The author should provide more information about Figure 5 volcano plot, what information can be extracted from the volcano plot?

**Reply:** We sincerely appreciate your detailed comments and suggestions. In **Lines 258-264 of the** **file labeled 'Revised Manuscript with Track Changes'** (with all modifications **highlighted in yellow**), we have supplemented essential information regarding the interpretation of the volcano plot.

**7. Comment:** Line 367. substantiates may be too strong for science, probably using indicates or suggests would be better.

**Reply:** We sincerely appreciate this insightful linguistic correction. In **Line 363 of the** **file labeled 'Revised Manuscript with Track Changes'** (**highlighted in yellow**), the verb "substantiates" has been replaced with "suggests" to more precisely convey the inferential nature of our findings.

**8. Comment:** Line 380. 'In line with our study,' please indicate which figures or tables are 'in line with' and how?

**Reply:** We sincerely thank you for your constructive suggestion. In **Lines 374-378 of the** **file labeled 'Revised Manuscript with Track Changes'** (with all modifications **highlighted in yellow**), we have revised the text to explicitly establish a connection between our data and the Th2-immunity mechanism, while providing figure-based evidence to support this linkage.

Once again, we extend our sincere gratitude to the reviewers and editors for their invaluable contributions. We believe that the manuscript has been significantly enhanced, and we look forward to its potential publication in your esteemed journal.

Sincerely,

Hua Zhang
